# Effect of low-dose rivaroxaban on bleeding events in low-weight patients with nonvalvular atrial fibrillation: a retrospective propensity score-matched cohort study

**DOI:** 10.3389/fcvm.2024.1495377

**Published:** 2024-11-13

**Authors:** Yuan Liu, Hengli Lai, Zhenhuan Chen, Ganwei Xiong

**Affiliations:** ^1^ Jiangxi Medical College, Nanchang University, Nanchang, China; ^2^ Department of Cardiology, Jiangxi Provincial People’s Hospital, The First Affiliated Hospital of Nanchang Medical College, Nanchang, China; ^3^ Department of Cardiology, The First People’s Hospital of Xiushui County, Jiujiang, China

**Keywords:** low body weight, rivaroxaban, nonvalvular atrial fibrillation, hemorrhage, low dose

## Abstract

**Objective:**

To investigate the effect of low-dose rivaroxaban on bleeding events in low-weight patients with nonvalvular atrial fibrillation.

**Methods:**

A retrospective study was conducted in patients with nonvalvular atrial fibrillation (weight ≤ 60 kg) who were admitted to the Department of Cardiology of Jiangxi Provincial People's Hospital from June 1, 2022, to December 12, 2022 and received anticoagulant therapy with rivaroxaban. The patients were divided into standard-dose group (15–20 mg) and low-dose group (10–15 mg). The patients were followed up for an average of 15 months by outpatient examination, telephone follow-up, or medical record inquiry of readmission patients. The bleeding events of the two groups were recorded during the follow-up period, and the two groups were balanced by propensity score weighting.

**Results:**

A total of 198 patients with NVAF and body weight ≤60 kg receiving rivaroxaban anticoagulation therapy were enrolled, including 65 patients in the standard-dose group (15–20 mg) and 133 patients in the low-dose group (10–15 mg). In this study, only 24.1% (65/241) of the patients followed the standard dose of rivaroxaban. There was no significant difference in the incidence of bleeding events between the two groups after the balance of baseline characteristics (age) (*P* > 0.05). This was also consistent in patients weighing less than 50 kg.

**Conclusions:**

In the real world, in lower-weight patients with nonvalvular atrial fibrillation, a reduced dose of rivaroxaban did not reduce the risk of bleeding, and this was consistent in patients weighing less than 50 kg.

## 1 Introduction

Asian patients with atrial fibrillation (AF) are less likely to receive a comprehensive rhythm control strategy, cardioversion, and atrial ablation (CA) compared to their American counterparts. They show a preference for oral anticoagulants (1), and low body weight is relatively common in Asian populations, which may impact the effectiveness of anticoagulation for AF (2–5). Several studies (4, 6, 7) have confirmed that direct oral anticoagulants (DOACs) show better efficacy and safety than warfarin in patients with low body weight, and have been widely used in the prevention of thromboembolic events in patients with nonvalvular atrial fibrillation. Although there is no need for routine monitoring of drug concentration for NOACs, it is still important to select the appropriate dose of new oral anticoagulants (NOACs) according to the dosing criteria defined in randomized controlled trials, especially for patients with low body weight.

The recommended dose of rivaroxaban for patients with nonvalvular atrial fibrillation is 20 mg·d-1(CrCl ≥ 50 ml·min-1) in the guidelines and consensus of other Asian countries and Europe except Japan (8–10). In the guidelines, the dose of rivaroxaban can be reduced to 15 mg·d-1 only for patients with CrCl = 30–49 ml·min-1. The guidelines only recommend that the dose be reduced to 15 mg·d-1 for patients with atrial fibrillation and low body weight, but the instructions did not mention whether the dose should be adjusted for patients with low body weight. Low-dose rivaroxaban (10 mg·d-1, 15 mg·d-1) is also widely used in Asian patients with nonvalvular atrial fibrillation despite insufficient evidence, but there are few studies on its safety, and the results are different. There is a consensus on the use of low-dose anticoagulants in clinical practice. However, for patients with low body weight, there is a lack of evidence-based medical evidence for the use of rivaroxaban in patients with low body weight except pharmacokinetics evidence. Moreover, patients cannot precisely break the drug in half and take it. Therefore, this study aims to link low body weight with a low dose of rivaroxaban. We collected the clinical data of 144 underweight patients with nonvalvular atrial fibrillation (NVAF) treated with rivaroxaban in our hospital, to investigate the effect of different doses of rivaroxaban on bleeding in these patients, and whether a reduced dose of rivaroxaban would reduce the risk of bleeding.

## 2 Data and methods

### 2.1 Study population

In this retrospective cohort study, we collected information on patients with nonvalvular atrial fibrillation (NVAF) who were treated with rivaroxaban in the Department of Cardiology of Jiangxi Provincial People's Hospital from June 1, 2022, to December 12, 2022, through the Chinese Atrial Fibrillation Center System. Following the rivaroxaban label, we screened patients who were off-label and contraindicated in a real-world setting. Inclusion criteria: (1) non-valvular atrial fibrillation; (2) regular use of rivaroxaban 10 mg·qd-1, 15 mg·qd or 20 mg·qd-1 within 1 year after discharge; (3) body weight ≤ 60 kg. Exclusion criteria: (1) Valvular atrial fibrillation; (2) patients who did not regularly take/changed to anticoagulant drugs within 1 year after discharge; (3) pregnant patients; (4) severe hepatic and renal dysfunction (Child-Pugh grade; Patients with CrCl < 15 ml/min); (5) patients with hematological or immune system diseases or coagulation dysfunction; (6) acute cardio-cerebrovascular accident within 6 months; (7) allergic to rivaroxaban or combined with severe allergic diseases; (8) unable to complete the follow-up due to their reasons. Non-valvular atrial fibrillation (NVAF) was defined according to Atrial Fibrillation: current understanding and management recommendations (2021). Regular use of rivaroxaban was defined as a proportion of days covered (PDC) ≥ 80%, the PDC is an objective measure used to accurately estimate adherence to medication for chronic diseases, defined as the ratio of the number of days a medication is available/days since the prescription fill date (11). PDC ≥80% was classified as adequate adherence to medication (12). Low body weight was defined as ≤ 60 kg and very low body weight as <50 kg. According to the instructions (10) of rivaroxaban, Chinese expert Recommendations (13) on the Clinical Application of Rivaroxaban and Chinese Guidelines for the diagnosis and treatment of Acute Ischemic Stroke (2018 edition) (14), the recommended dose of rivaroxaban for patients with non-valvular atrial fibrillation is 20mg·qd (CrCl ≥ 50 ml·min-1). 15 mg·d-1(CrCl = 15–49 ml·min-1). For patients with NVAF, there was no evidence of the use of rivaroxaban at doses less than 10 mg, so doses less than 10 mg were not included in this study.

### 2.2 Information collection

The general clinical data and laboratory data of patients during hospitalization could be searched through the atrial fibrillation center and the electronic medical record management system of our hospital. After discharge, the patients were followed up by outpatient examination, telephone interview, or through the AF center system for patients with re-admission during follow-up. According to the different doses used, the patients were divided into the standard-dose group (15–20 mg) and the low-dose group (10–15 mg). The patient's age, gender, weight, CHA_2_DS_2_-VASc score, HAS-BLED score, combined diseases, drug treatment, and other clinical data were collected. The patients were followed up for an average of 15 months. The bleeding and death events during the follow-up period were recorded and compared between the two groups to explore the effects of different doses of rivaroxaban on patients with low body weight.

Subgroup analysis: According to body weight, the patients were divided into a low body weight group (50–60 kg) and a very low body weight group (<50 kg) to explore the effect of different doses of rivaroxaban on very low body weight patients.

### 2.3 Clinical outcomes

Please refer to the definition of the International Society on Thrombosis and Haemostasis(ISTH) (15) for grading bleeding events during OAC therapy: (1) Major bleeding includes fatal bleeding, symptomatic bleeding in critical organs such as intracranial hemorrhage, intraspinal, intraocular, retroperitoneal, intraarticular, pericardial or intermuscular compartment syndrome; or bleeding resulting in a decrease of hemoglobin by more than 20 g/L or requiring infusion of at least 2 units of erythrocytes. (2) Clinically relevant non-major bleeding does not meet the criteria for major bleeding but necessitates hospitalization for medical intervention. (3) Minor bleeding refers to other types of bleeding.

### 2.4 Statistical analysis

**Figure 1:**
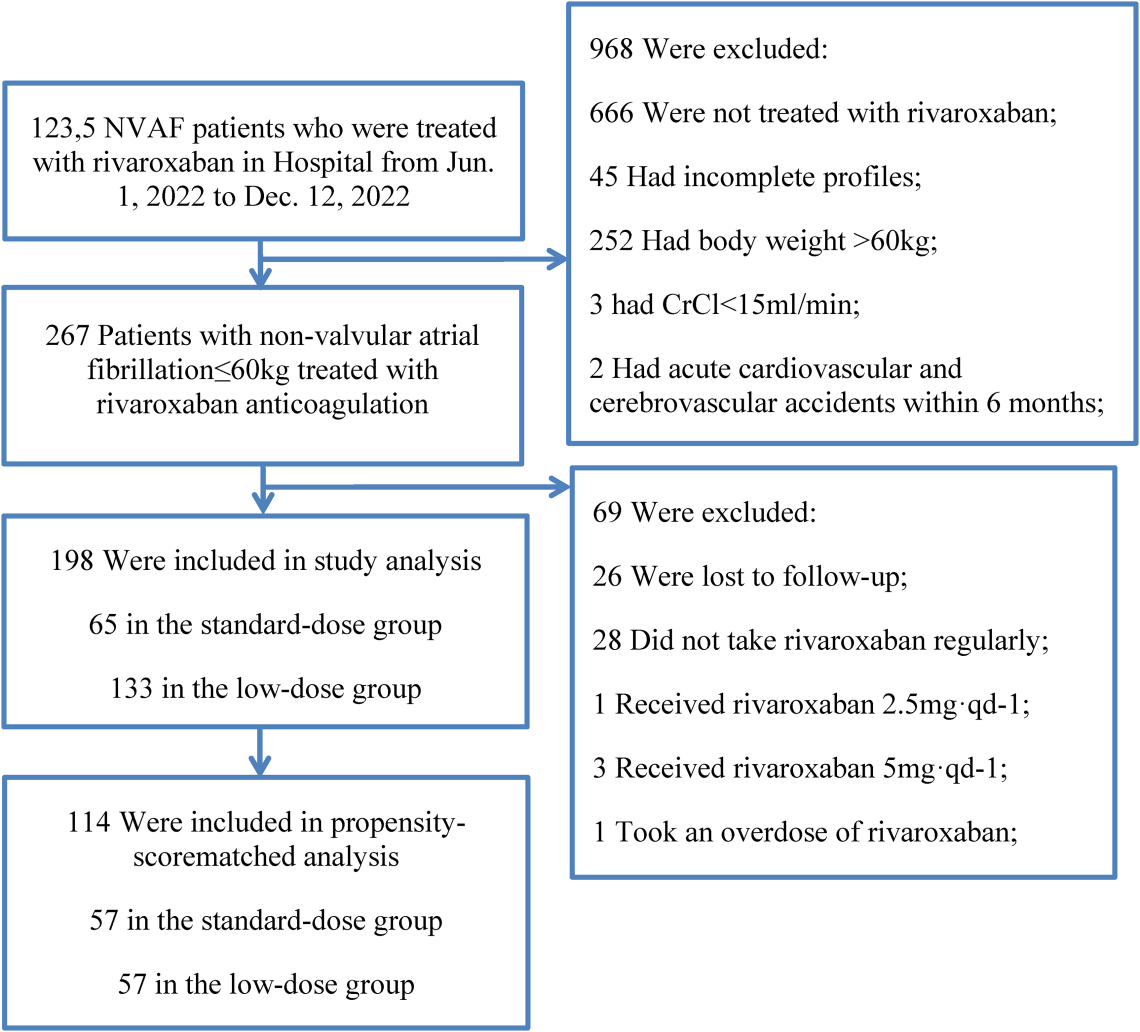
Flow chart of the study.

R Studio 4.3.1 statistical software was utilized for data analysis. Quantitative data underwent normality testing using the Shapiro-Wilk test, with normally distributed continuous variables presented as mean ± standard deviation (X ± S), non-normally distributed continuous variables expressed as median and interquartile range, and count data reported as number of cases and percentage (%). Statistical significance was assessed using *χ* test for categorical variables, the *t*-test for continuous variables, and the nonparametric rank-sum test for non-normally distributed continuous variables to compare differences between groups. *P* < 0.05 was considered statistically significant. In the real-world setting, numerous confounding factors can impact outcome events (bleeding events). Due to the differences in baseline characteristics between the two groups (as shown in Figure 1), propensity-score matching was utilized to identify cohorts of patients with similar baseline characteristics. Low-dose rivaroxaban was utilized as the dependent variable, with all baseline characteristics outlined in Figure 1 serving as covariates. Matching was based on the estimated propensity scores using nearest-neighbor matching (1:1) within a caliper width of 0.2 standard deviations without replacement. Standardized differences in all baseline covariates were calculated before and after matching to evaluate prematch imbalance and postmatch balance (16). Standardized differences of less than 10.0% indicate a small imbalance (16).

## 3 Results

### 3.1 General conditions of NVAF patients treated with rivaroxaban in standard-dose group (15–20 mg) and low-dose group (10–15 mg)

A total of 267 patients with non-valvular atrial fibrillation were followed up, 26 patients were lost to follow-up, 28 patients did not take rivaroxaban regularly, 1 patient received rivaroxaban 2.5 mg·qd-1, 3 patients received rivaroxaban 5 mg·qd-1, 1 patient took an overdose of rivaroxaban. A total of 198 patients were enrolled in the study, and 27 bleeding events occurred. There were 65 patients in the standard-dose group and 133 patients in the low-dose group (Table 1). The coincidence rate between the clinical dose of rivaroxaban and the standard dose of rivaroxaban in this study was only 24.1% (65/241) (214 patients included those who used rivaroxaban irregularly or with the wrong dose).

**Table 1:** Baseline data in the standard-dose group and the low-dose group.

Characteristic	Before matching				After matching			
	Standard dose (*n* = 65)	Low dose (*n* = 133)	*P*	Standardized difference, %	Standard dose (*n* = 57)	Low dose (*n* = 57)	*P*	Standardized difference, %
Men (%)	21 (32.31)	61 (45.86)	0.069	29.0	20 (35.09)	19 (33.33)	0.843	3.7
Age (years)	71.60 ± 8.40	75.65 ± 9.27	0.003	48.3	72.05 ± 8.75	72.40 ± 7.95	0.823	4.0
Height (cm)	158.00 (150.00, 164.00)	158.00 (152.00, 162.00)	0.63	6.4	158.00 (151.00, 164.00)	157.00 (152.00, 160.00)	0.914	5.8
Weight (kg)	51.00 (49.00, 55.00)	51.30 (47.00, 56.00)	0.808	6.7	51.00 (49.00, 56.00)	51.30 (47.00, 56.00)	0.776	8.1
50–60	46	86			40	35		
<50	19	47			17	22		
BMI (kg/m^2^)	20.50 (19.30, 22.90)	20.70 (19.50, 22.50)	0.785	1.1	20.50 (19.10, 22.90)	20.70 (19.60, 22.40)	0.872	1.9
CrCl (ml/min)	45.30 (37.10, 57.50)	50.40 (37.60, 60.80)	0.356	6.9	45.30 (37.00, 59.70)	50.70 (39.50, 59.30)	0.449	2.0
≥50	22	69			21	30		
<50	43	64			36	27		
CHA2DS2-VASc score	4.00 (3.00, 5.00)	4.00 (3.00, 5.00)	0.68	7.5	4.00 (3.00, 5.00)	4.00 (3.00, 5.00)	0.721	5.6
1–2	10	17			10	9		
≥3	55	116			47	48		
HAS-BLED score	1.00 (1.00, 2.00)	1.00 (1.00, 2.00)	0.474	10.6	1.00 (1.00, 2.00)	2.00 (1.00, 2.00)	0.66	7.7
0–2	56	117			48	49		
≥3	9	16			9	8		
Hypertension (%)	42 (64.62)	70 (52.63)	0.11	25.1	34 (59.65)	33 (57.89)	0.849	3.6
Diabetes mellitus (%)	9 (13.85)	24 (18.05)	0.457	12.2	8 (14.04)	7 (12.28)	0.782	5.1
Coronary disease (%)	11 (16.92)	28 (21.05)	0.493	11.0	10 (17.54)	9 (15.79)	0.802	4.6
Heart failure (%)	47 (72.31)	107 (80.45)	0.196	18.2	41 (71.93)	42 (73.68)	0.833	3.9
Previous stroke (%)	12 (18.46)	27 (20.30)	0.76	4.7	11 (19.30)	11 (19.30)	1	0.0
COPD (%)	5 (7.69)	22 (16.54)	0.088	33.2	5 (8.77)	6 (10.53)	0.751	6.2
use antiplatelet drug (%)	13 (20.00)	23 (17.29)	0.643	6.8	11 (19.30)	12 (21.05)	0.815	4.4
Previous major bleeding (%)	2 (3.08)	5 (3.76)	1	4.0	2 (3.51)	1 (1.75)	1	9.5

There were no significant differences between the two groups in gender, height, weight, BMI, CHA_2_DS_2_-VASc score, HAS-BLED score, CrCl, past medical history (hypertension, diabetes, coronary heart disease, chronic heart failure, major bleeding, ischemic stroke, TIA, chronic obstructive pulmonary disease), or previous concomitant medications (antiplatelet drugs) (Table 1). Patients in the standard-dose group were younger overall than those in the low-dose group (71.60 ± 8.40 vs. 75.65 ± 9.27 years, *P* = 0.003). The standard difference rate of each index before matching was 4.0%–48.3%. After matching, the standard difference rate was controlled within 10.0% (Table 1).

### 3.2 Bleeding events in the rivaroxaban standard-dose group vs. low-dose group

The average follow-up of 15 months showed that there were 7 cases of bleeding events in the standard-dose group (7/65, 10.8%), and 20 cases of bleeding events in the low-dose group (20/133, 15.0%) (Table 2). The most frequent bleeding events were minor bleeding (71.4%, 55.0%) in both groups. After propensity score matching to balance the differences in clinical characteristics between the two groups, the results showed that there was no significant difference in the incidence of bleeding events between the two groups (*P* > 0.05) (Table 2).

**Table 2:** Bleeding events in the rivaroxaban standard-dose group as compared with the low-dose group.

	Before matching		After matching			
	Standard dose (*n* = 65)	Low dose (*n* = 133)	Standard dose (*n* = 57)	Low dose (*n* = 57)	*P*	OR (95% CI)
Bleeding events, *n* (%)	7 (10.8)	20 (15.0)	5 (8.8)	6 (10.5)	0.739	0.80 (0.21–2.98)
Major bleeding	0	2	0	0		
Clinically relevant non-major bleeding	2	7	1	3	0.341	0.33 (0.03–3.20)
Minor bleeding	5	11	4	3	0.657	1.50 (0.25–8.98)

### 3.3 Subgroup analysis according to weight stratification of 50–60 kg and <50 kg

After an average follow-up of 15 months, for patients weighing 50–60 kg, there were 6 bleeding events (6/46, 13.0%) in the standard-dose group and 10 bleeding events (10/84, 11.9%) in the low-dose group (Table 3). After propensity score matching to balance the differences in clinical characteristics (age) between the two groups, there was no significant difference in bleeding events between the two groups (*P* > 0.05) (Table 3). For patients <50 kg, there was 1 case (1/19, 5.2%) of bleeding events in the standard-dose group and 10 cases (10/47, 21.3%) in the low-dose group (Table 3). There was no significant difference in baseline characteristics between the two groups, and there was no significant difference in bleeding events between the two groups after propensity score matching (*P* > 0.05) (Table 3).

**Table 3:** Bleeding events, stratified according to body weight of 50–60 kg versus less than 50 kg.

		Before matching		After matching			
		Standard dose (*n* = 46)	Low dose (*n* = 84)	Standard dose (*n* = 33)	Low dose (*n* = 33)	*P*	OR (95% CI)
50–60 kg (*n* = 132)	Bleeding events, *n* (%)	6 (13.0)	10 (11.9)	3 (9.0)	6 (18.2)	0.327	0.50 (0.13–2.00)
		Before matching		After matching			
		Standard dose (*n* = 19)	Low dose (*n* = 47)	Standard dose (*n* = 12)	Low dose (*n* = 12)	*P*	OR (95% CI)
<50 kg (*n* = 66)	Bleeding events, *n* (%)	1 (5.2)	10 (21.3)	1 (8.3)	1 (8.3)	>0.999	1.00 (0.06–15.99)

## 4 Discussion

Although clinicians tended to use a lower dose of rivaroxaban than the standard dose (133/198, 67.2%) in low-weight (≤60 kg) patients with nonvalvular atrial fibrillation, regardless of whether renal function was normal or not, there was no statistically significant difference in bleeding events in the lower-dose (10–15 mg) group, suggesting that a reduced dose of rivaroxaban does not reduce the risk of bleeding. Even for very low body weight (<50 kg), there was no need to reduce the dose of rivaroxaban. The possible reasons for this conclusion are as follows: (1) The age of the low-dose group is generally higher than that of the conventional-dose group, and the elderly are often complicated with frailty, malnutrition, and a variety of complications, which may affect the anticoagulation effect. (2) Many previous studies reported that a high interindividual variability in the drug blood levels was shown with all DOACs, and showed an association between DOAC plasma levels and bleeding complications during follow-up (13, 14, 17–19). Moreover, an observational, multicenter study proposed that bleeding complications during DOAC treatment were more frequent among NVAF patients with higher C-peak anticoagulant levels (20). (3) After matching, the proportion of combined antiplatelet drugs in the low-dose group was higher than that in the standard-dose group. Several studies have suggested that the risk of bleeding is greater with the combination of anticoagulation and antiplatelet therapy than with anticoagulation alone (21–23). (4) Radiofrequency ablation was performed in 21.5% (44/198) of the patients in our study, and catheter ablation should be performed during uninterrupted oral anticoagulation. Procedures such as vascular puncture may increase the risk of bleeding. However, in the biggest real-life data registry regarding CA ablation on rivaroxaban in an Italian setting (IRIS) (*n* = 250), only one minor bleeding event was recorded, and no major bleeding event was recorded during 12 months of follow-up (24). (5) eGFR is a dynamic indicator, and patients’ renal function may improve or worsen during the follow-up period, which may cause information bias and affect the results. (6) The follow-up time was too short, and the bleeding events were not accurately observed.

In a subgroup analysis (25) of a national observational study in Korea, the net clinical benefit of standard-dose DOACs compared with low-dose DOACs in AF patients ≤60 kg was almost all neutral, which is consistent with the current findings. Hamatani et al. showed no difference in the rate of major bleeding in patients with low body weight (≤50 kg) compared with those with >50 kg in their analysis of data from the prospective, community-based Japanese Fushimi AF Registry, but that study was based on all DOACs and did not further explore patients receiving low-dose anticoagulation. At the same time, several studies (26–28) have found that in Asian patients, low-dose rivaroxaban has a similar risk of bleeding, but the incidence of embolic events is significantly higher, but the type of embolic events is different in different studies. Due to the retrospective study, 22.2% (44/198) of patients underwent radiofrequency ablation or left atrial appendage closure and were isolated during the COVID-19 epidemic. Embolism and death events could not be accurately counted, and only association evidence rather than causality evidence was provided. In conclusion, there is no unified conclusion on the efficacy and safety of low-dose rivaroxaban in low-weight patients with nonvalvular atrial fibrillation, and larger multicenter prospective studies are still needed to provide evidence for clinical treatment.

## 5 Limitation

This study has several limitations. It was conducted in a single center and had a relatively small sample size. Future large-scale multicenter randomized studies comparing different body weight stratification are warranted.

## 6 Conclusion

In underweight patients with nonvalvular atrial fibrillation, adherence to the guideline-recommended dose of rivaroxaban was low, and the reduced dosage did not decrease the risk of bleeding, a finding that remained consistent among patients weighing less than 50 kg. However, there are still some limitations in this study, such as a small sample size, insufficient observation indicators, and lack of embolic events, which need to be further explored and verified by larger prospective studies.

## Data Availability

The raw data supporting the conclusions of this article will be made available by the authors, without undue reservation.
